# The antifungal mechanism of EntV-derived peptides is associated with a reduction in extracellular vesicle release

**DOI:** 10.1371/journal.ppat.1013519

**Published:** 2025-09-22

**Authors:** Giuseppe Buda De Cesare, Melissa R. Cruz, Shane A. Cristy, Luis A. Vega, Robert Zarnowski, Antonino Zito, Shantanu Guha, David R. Andes, Danielle A. Garsin, Michael C. Lorenz

**Affiliations:** 1 Department of Microbiology and Molecular Genetics, University of Texas McGovern Medical School, Houston, Texas, United States of America; 2 Department of Medical Microbiology and Immunology, University of Wisconsin, Madison, Wisconsin, United States of America; 3 Department of Molecular Biology, Massachusetts General Hospital, Boston, Massachusetts, United States of America; 4 Department of Genetics, The Blavatnik Institute, Harvard Medical School, Boston, Massachusetts, United States of America; University of California Irvine, UNITED STATES OF AMERICA

## Abstract

*Candida albicans*, an opportunistic fungal pathogen, causes systemic and superficial infections, especially in immunocompromised patients. Treatment of fungal infections is complicated by limited antifungal options and the development of drug resistance. Previous work from our group demonstrated the efficacy of the anti-virulence peptide EntV and shorter variants against *C. albicans* infection in various animal models, including mouse models of oropharyngeal candidiasis and disseminated infection and a rat venous catheter model. However, the mechanism of action, which abrogates fungal virulence without fungicidal or fungistatic activity, has remained unknown. We used a combination of cell biological, biochemical, genomic, and genetic approaches to identify this mechanism. We demonstrate that EntV-based peptides bind to the fungal cell envelope in a punctate and dynamic manner, co-localizing with extracellular vesicles (EVs), which play a critical role in fungal biofilm formation and virulence. Transcriptomic and genetic analyses further indicate that this activity is linked to the intracellular vesicular trafficking machinery, especially the ESCRT pathway, as mutations in this pathway alter sensitivity to EntV peptides and regulate virulence. We also show that EntV treatment significantly reduces EV secretion in *C. albicans*, supporting a novel mechanism of antifungal action through inhibition of EV-mediated virulence. These findings further develop EntV as a promising anti-virulence agent with potential for therapeutic development against drug-resistant fungal pathogens.

## Author summary

There is a desperate need for novel antifungal drugs to augment the few approved options. We have identified a bacterial protein, EntV, that has profound, if unusual, antifungal properties and previous work has reduced this protein to a 12 amino acid peptide that is effective in several animal models of infection with *Candida* *albicans*, the most common fungal pathogen. We show here that this EntV 12mer binds to the cell surface of *C*. *albicans* and other species in a punctate and dynamic pattern that resembles extracellular vesicles (EVs), secreted organelles that are known to play important roles in host-pathogen interactions. Using genetics and transcriptomics, we identify a series of genes responsive to EntV treatment that are involved in EV secretion. Mutations in these genes alter susceptibility to the peptide and generally reduce virulence in animal models. Treatment with EntV inhibits EV secretion and those vesicles are more irregular. Together, we propose a novel antifungal mechanism that does not rely on static or cidal effects but rather alters the release of host-stimulatory EVs to attenuate virulence.

## Introduction

Fungal infections represent a growing burden on the healthcare system [1,2]. In the United States alone, the direct and indirect medical costs for such infections are estimated at over $10 billion annually (Centers for Disease Control and Prevention, 2024). Moreover, fungal diseases can further exacerbate other chronic conditions such as diabetes [3], asthma [4], and cystic fibrosis [5]. The incidence of serious fungal infections has risen for many years, the result of an increase in the population of susceptible patients due to immunocompromise or other debilitating conditions. At the same time, emerging or previously rare species are becoming more common; increased prophylaxis, agricultural use of azoles, and climate change have been mooted as potential causes. Despite this, *Candida, Aspergillus*, and *Cryptococcus* remain the clinically dominant genera. Additionally, the treatment of such infections is hindered by limited antifungal options and the development of drug resistance [6]. Currently, the arsenal of antifungals available for clinical use on systemic fungal infections consists of only three classes, namely echinocandins, azoles, and polyenes. Despite active development [7], the only approved antifungal agents in the last two decades belong to the previously mentioned classes, including isavuconazole [8], oteseconazole [9], and rezafungin [10]. Ibrexafungerp, a β-glucan synthase inhibitor structurally unrelated to echinocandins, was recently approved to treat recurrent vulvovaginal candidiasis [11]. The limited structural diversity of molecules as well as their mode of action exacerbate the emergence of drug-resistant isolates, complicating the treatment of these diseases and posing additional threats to the current antifungal armamentarium [12]. The increase in azole resistance among *Candida* and *Aspergillus* species is one of the greatest challenges [13], as well as the emergence of multidrug-resistant strains like *Candida auris* and the *Mucorales spp.* [14,15]. Another drawback of the current antifungals includes the safety profile, particularly hepatotoxicity and nephrotoxicity, which occur commonly in patients treated for systemic fungal infections [16].

One of the most common fungal pathogens, *Candida albicans*, is a commensal and part of the healthy human microbiota [17]. Its metabolic plasticity and adaptation to different environmental conditions allow this fungal organism to colonize several body niches, including but not limited to, mucosal surfaces of the gastrointestinal (GI) and genitourinary tracts, and the oral cavity [18,19]. However, alterations of the host microbiota and immune system, such as the use of antibiotics or immunosuppressant therapies, highlight the opportunistic nature of this organism, which causes infections ranging from superficial (such as oral thrush and vaginitis) to systemic [18,20]. Over 1.5 million people suffer from *Candida* bloodstream infections every year, with a mortality rate between 35–40% worldwide, which rises to 90% if left untreated [1,21].

The potential for interspecies interactions to shape the microbiota and influence the progression of infection is largely unexplored. However, consequential relationships have been described between *C. albicans* and *Staphylococcus aureus, Pseudomonas aeruginosa*, and *Streptococcus* species. Whether these interactions involve secreted mediators that may be developed into novel antimicrobial agents that could be clinically exploited is even less well explored. We have described one such pairing, between *C. albicans* and *Enterococcus faecalis*. This Gram-positive bacterium shares the same niches with *C. albicans*, such as the oral cavity, GI, and urogenital tracts [22,23], and is frequently isolated with *C. albicans* in clinical specimens [24]. In some circumstances, though, they appear to promote a commensal association with the host [25–28], and subsequent work demonstrated that *E. faecalis* antagonizes *C. albicans* virulence through a mechanism mediated by EntV, a 68aa bacteriocin [27,29,30]. Based on structural data, a 12 amino acid fragment of EntV (hereafter, the ‘12mer’) was found to be fully active in both in vitro and in vivo experiments, including mouse models of oropharyngeal candidiasis, disseminated infection, and a rat venous catheter model [31]. Moreover, peptide variants with improved antifungal properties were also identified [32]. They were effective against drug-resistant strains of *C. albicans* and other fungal species, including *C. auris*, *Cryptococcus gattii, and C. neoformans* [31]. Notably, the antifungal activity of EntV consists of inhibition of adhesion and biofilm formation, without affecting cell viability or growth [30]. Unusually for an antimicrobial peptide, the 12mer is hydrophobic and has significant sequence flexibility [32]. EntV thus represents a unique anti-virulence agent.

In this study, we used a combination of cell biological, biochemical, genomic, and genetic approaches to identify the molecular mechanism by which EntV exerts antifungal effects. We show that the 12mer peptide derived from EntV binds to the cell envelope of *C. albicans* in a punctate and dynamic manner*,* forming multimers that lack a specific stoichiometry, also differentiating it from canonical antimicrobial peptides [33]. The 12mer colocalizes with a dye that preferentially labels extracellular vesicles (EVs) in both *C. albicans* and *C. gattii*. EVs are involved in virulence and biofilm formation in multiple fungal species [34–36]; therefore, targeting these vesicles is a plausible mechanism for EntV disruption of fungal virulence. A transcriptome analysis highlighted the involvement of a small group of genes connected to vesicular trafficking and a larger genetic analysis confirms a role for the ESCRT pathway in EntV sensitivity and virulence. Consistent with this, *C. albicans* cells treated with the 12mer release significantly fewer EVs than untreated cells. In summary, we herein show that the anti-virulence properties of EntV-derived peptides are likely exerted through inhibition of EV secretion.

## Results

### EntV peptides bind to the fungal cell surface in a punctate manner

**Fig 1:**
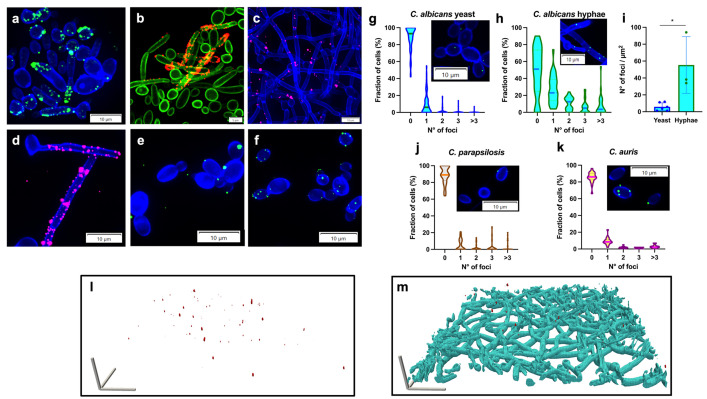
EntV peptides bind to the cell surface. Fluorescence microscopy of *C. albicans* SC5314 labeled with: (a) 1 µM EntV^68^-FITC and (b) EntV^68^ detected using anti-EntV^68^ [37] and visualized with an Alexa Fluor-594 secondary antibody. (c-d) 1 µM 12aa-Alexa-Fluor-647 was used to label *C. albicans* (c) SC5314 or (d) WO-1. 1 µM 12aa-Alexa-fluor-488 was used to label *Candida parapsilosis* (e), *Candida auris* (f). The cell wall was stained with calcofluor white in a, c, d, e, and f, and the plasma membrane labeled with Pma1-GFP in b**.** Foci distribution of *C. albicans* yeast, (g), and hyphae, (h), *C. parapsilosis*, (j), *C. auris*, (k), stained with the 12aa-Alexa Fluor-488. (i) Number of 12aa-Alex-Fluor-488 foci normalized by the surface area unit (µm2) of different *C. albicans* cell morphologies. Statistical significance was determined using a Wilcoxon Rank Sum Test. *P < 0.05. (l-m) BiofilmQ 3D rendering of a biofilm stained with 12aa-Alexa Fluor-647 peptide (l, pseudocolored in red) superimposed on the *C. albicans* hyphae (m, pseudocolored in light blue) highlights the binding of the peptide throughout the biofilm. Scale bars represent 20 µm in the x, y, and z directions. The Z-stacks were acquired using an Olympus IX-83 microscope as described in the Materials and Methods section.

Previous work showed EntV^68^ and the 12mer had similar anti-virulence activity against *C. albicans* biofilm formation in vitro and against *C. albicans* infection in both nematode and mouse models [31,32]. To begin interrogating the mechanism of action, the sub-cellular localization of the peptides was investigated using both fluorescently tagged peptides and immunofluorescent staining (Fig 1). The full length EntV^68^ was labelled with fluorescein isothiocyanate (FITC) at the C-terminus and incubated with yeast and hyphae of *C. albicans* for 2h, exhibiting a punctate staining pattern that was localized to the cell surface (Fig 1a). The same punctate pattern was observed using immunofluorescence staining employing a previously developed anti-EntV^68^ polyclonal antibody [37] (Fig 1b, controls in S1 Fig). To rule out that tagging may alter activity, the labeled peptide was tested in the *C. elegans* infection model, confirming that it is fully active (S2 Fig).

Similarly, the 12mer tagged with Alexa Fluor-647 displayed a punctate binding pattern (Fig 1c) resembling that of tagged EntV^68^ (Fig 1a). Moreover, the 12mer appeared to bind similarly to other *C. albicans* strains, such as the well-characterized WO-1, a strain studied for decades for its ability to undergo White-Opaque switching (Fig 1d), and other species, including *C. parapsilosis* and *C. auris* (Fig 1e and 1f, respectively). From live imaging experiments where live *C. albicans* cells were analyzed, the peptide binding pattern was highly dynamic, with puncta appearing and disappearing along the surface of fungal cells (S1 and S2 Movies). The peptides appeared to preferentially bind hyphal cells (Fig 1a, 1b, and 1d). To confirm this qualitative observation, we quantified the focal distribution (Fig 1g-1k). After acquiring Z-stack images, the number of foci was counted for each cell, with septa, visualized by calcofluor white staining, delineating one hyphal unit. Hyphal cells exhibited a higher number of foci per cell: 52% displayed at least one focus (Fig 1h), compared to 11% of the yeast cells (Fig 1g). Given the size difference between the two morphologies, we normalized the number of foci per unit of surface area of each cell using CellProfiler (Fig 1i). *C. auris* and *C. parapsilosis* yeast cells did not show any significant variation in the number of foci per cell compared to SC5314 (Fig 1j and 1k), except for a slight increase of cells with >3 foci in *C. auris* (Fig 1k). By this analysis, greater binding of the peptide to hyphae relative to yeast was again observed. Finally, to better highlight the surface binding of EntV peptides, SC5314 hyphae stained with calcofluor white and 12mer tagged with Alexa Fluor-647 were 3D rendered via BiofilmQ [38] (Fig 1l and 1m and S3 Movie). This experiment confirms, once again, the superficial localization of the EntV foci on *Candida* cells.

Together these data suggest that the target of binding is localized on the cell surface, and is more abundant on the filamentous form of *C. albicans*, but conserved in related species.

### Binding of the peptide to the cell surface is necessary, but not sufficient, for activity

**Fig 2:**
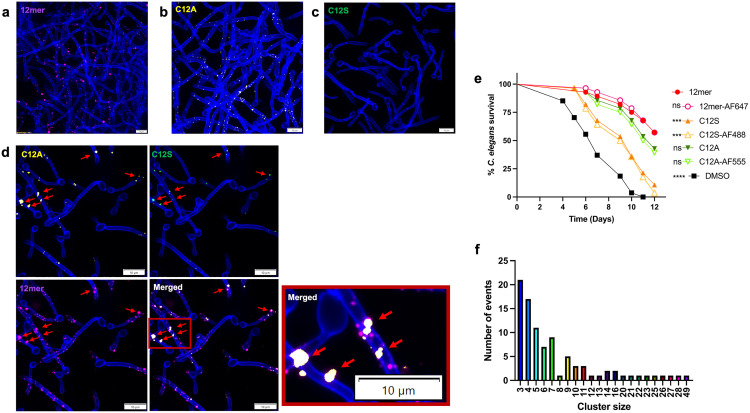
Cysteine substitutions have different effects on 12mer activity/staining pattern. Fluorescence microscopy of *C. albicans* hyphae incubated with 1µM: (a) wild type 12mer-Alexa Fluor-647, (b) C12A variant, (c) C12S variant. (d) *C. albicans* cells stained with calcofluor white (blue) and labeled with a mix of wild-type 12mer-Alexa Fluor-647 (purple), C12A variant-Alexa Fluor-555 (yellow), and C12S variant-Alexa Fluor-488 (green), highlighted by the red arrows. (e) Survival of *C. elegans* infected with *C. albicans* and exposed to 1nM of the indicated peptides with statistical significance in comparison to the 12mer control determined by Mantel-Cox log-rank analysis. ***P < 0.001, ****P < 0.0001. (f) Cumulative distribution of STochastic Optical Reconstruction Microscopy (STORM) of *C. albicans* hyphae incubated with 1µM 12mer peptide labeled with Alexa Fluor-647.

The active 12mer contains a C-terminal cysteine critical for activity [31]. In our previous work, not including the cysteine in the peptide and/or changing the peptide to serine reduced efficacy in a nematode model of infection in which the WT 12mer is highly protective [31]. To test if the cysteine is required for binding to the cell envelope, we synthesized variants in which this residue was substituted with either serine, a polar residue of similar size, or alanine, a smaller neutral residue, and incubated with *C. albicans* cells (Fig 2). The C12A variant displayed a punctate binding pattern similar to the wild-type peptide (Fig 2a and 2b), whereas C12S did not bind (Fig 2c). To test if these binding results were congruent with their level of activity, they were also assayed in the *C. elegans* infection model of candidiasis. The fluorescently-tagged serine variant was severely attenuated in protecting nematodes from fungal killing, as we previously reported for the unlabeled peptide [32]. However, C12A protected *C. elegans* as well as the original peptide (Fig 2e). Interestingly, the inability of the serine variant to bind the cell surface could be rescued by incubation with the wild type or the C12A variant peptides; colocalization of all three peptides within the same foci was observed (Fig 2d). In all cases, C-terminal labeling with a fluorophore did not affect antifungal activity (Fig 2e). It is notable that the C12S peptide, a substitution generally thought to be more conservative than C12A, had a more severe phenotype in activity and surface binding.

We used Matrix-Assisted Laser Desorption/Ionization Time-of-Flight (MALDI-TOF) mass spectrometry analysis to assess the multimerization status of the 12mer. We found that it preferentially forms dimers in solution. In contrast, the C12S and C12A peptides are predominantly monomers, suggesting disulfide bond formation in the WT peptide; because the C12A is active, disulfide formation is not necessary and may be an artifact of these experimental conditions (S3 Fig). To assess whether the 12mer forms oligomeric complexes in vivo consistent with pore formation, we used Stochastic Optical Reconstruction Microscopy (STORM) to optically isolate single 12mer molecules to determine macromolecular clustering and if there was oligomerization at a specific stoichiometry. The peptide clusters were identified using a density-based spatial clustering of applications with noise (DBSCAN) algorithm [39]. As the 12mer typically forms dimers in solution, we defined clusters as a minimum of three peptide molecules within a 50 nm range. The cumulative distribution of clusters resulting from the analysis is shown in Fig 2f. As expected, the 12mer oligomerized on the cell surface of *Candida*, but the number of molecules per cluster ranged from 3 to 49. Therefore, no specific stoichiometry appears to be required for peptide activity. However, smaller aggregates (i.e., 3–7 molecules) were more abundant (Fig 2f). We conclude that the size of the peptide and the small aggregates is inconsistent with a pore-forming mechanism.

### Evidence for EntV peptides binding extracellular vesicles

**Fig 3:**
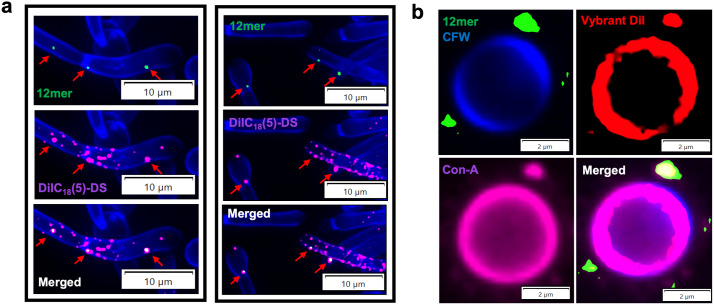
Extracellular vesicle staining colocalizes with 12mer peptide. (a) Fluorescence microscopy of *C. albicans* cells labeled with 1µM 12mer-Alexa Fluor-488 (green) and stained with calcofluor white (blue) and DiIC18 [5]-DS (purple). (b) Fluorescence microscopy of *C. gattii* labeled with 1 µM 12mer-Alexa Fluor-488 (green) and stained with calcofluor white (blue), concanavalin A (purple) and Vybrant DiI (red).

In addition to still photography, we collected live videos of *C. albicans* hyphae incubated with EntV^68^-FITC. In the videos, the punctate binding was very dynamic with fluorescent foci appearing and disappearing on the surface of the cells (S1 Movie). While there are several discrete structures on the membrane and cell wall of *C. albicans*, including lipid rafts, exposed β-glucan, actin patches, and others, initial assessment with markers of these structures was negative. Instead, the punctate and dynamic localization pattern was potentially consistent with extracellular vesicle (EV) release. As EVs are known to mediate host-pathogen interactions and biofilm formation, we tested the hypothesis that EntV peptides bind to EVs by performing co-localization studies with the dialkylcarbocyanine probe DiIC_18_ [5]-DS (‘DilC’), which was previously shown to localize to EVs in fungi preferentially [40]. Using fluorescent confocal microscopy, a punctate pattern of staining to the cell surface was observed with DilC, consistent with that expected for EVs [40] (Fig 3a). We observed that the 12mer peptide colocalized with a subset of EVs as visualized with the DilC dye, and was rarely seen in foci separate from DilC localization. (Fig 3a). Since these structures were first described and characterized mainly in *Cryptococcus* [41], a strain of *C. gattii* was used to assess the colocalization of the peptide with EVs (Fig 3b). The colocalization observed for *Candida* was also seen in *Cryptococcus*, where the peptide associated with another lipophilic dye, Vybrant DiI, as well as the lectin concanavalin A. This lectin stains the sugars, mainly in the mannan layer of the cell wall, which was previously shown to be present on the surface of EVs [42]. The colocalization of the peptide with concanavalin A demonstrates the presence of cell wall material on the binding target of EntV, supporting the hypothesis of the peptide localizing to EVs.

### The transcriptional response to EntV peptides identifies genes with potential roles in vesicle trafficking

To explore how EntV peptides might alter host interactions, we explored the transcriptional response under conditions where the peptide alters *Candida* physiology. Specifically, we used artificial saliva medium [30,43], a host-like environment where the peptide inhibits adhesion and biofilm formation [30,31,44]. *C. albicans* strain SC5314 was incubated with either the full-length 68mer, 12mer, or DMSO as vehicle control for 4, 24, and 48 hours on polystyrene plates in saliva medium at 37°C, and total RNA from multiple biological replicates was isolated and sequenced.

**Fig 4:**
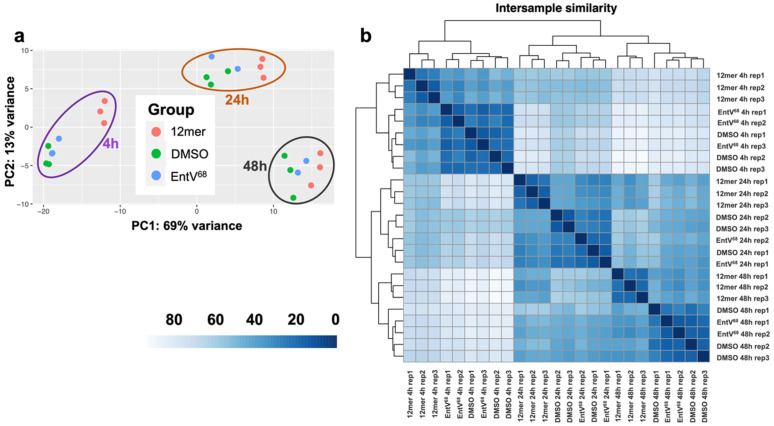
Transcriptional response of EntV-treated *C. albicans* cells. (a) Principal component analysis of the RNA sequencing data of *C. albicans* cells treated with DMSO, EntV^68^, and 12mer at 4, 24, and 48h in artificial saliva medium. (b) Intersample similarity of the RNA sequencing samples. The darker the color, the more similar the transcriptional response between samples.

Analysis of the RNA-seq data suggested the impact of EntV or the 12mer on the transcriptional response was limited. Principal component analysis (PCA) revealed that gene expression correlated more strongly with the time points rather than the treatment (Fig 4a). To explore further the correlation between samples, an intersample similarity matrix was created (Fig 4b). The analysis further confirmed what was observed in the PCA; there was greater similarity based on time point rather than treatment. Because the differences based on timepoints were likely impacted by the stage of biofilm development and the media’s nutritional content, we prioritized the modest impact of the treatment, as shown in S2 Table. The 12mer peptide altered the expression of only six genes compared to the control group, and these effects were greater than with EntV^68^ (Fig 4b). Table 1 shows the log_2_ fold-change values in the small subset of genes that were affected at the early time points (4 and 24h) when the changes were most distinct (Tables 1 and S2). Given the effects of the 12mer on biofilm formation and virulence, our observation that so few genes are transcriptionally affected may be consistent with a post-translational mechanism; altering the abundance or function of EVs is congruent with these findings.

**Table 1:** Genes differentially regulated in the presence of EntV peptides. Genes found differentially expressed (log2 fold change >4) between the DMSO control and EntV-treated samples in *C. albicans* cells grown in artificial saliva medium at 4, 24, and 48 h. ND indicates that the transcript was not detected in that condition. Descriptions are taken from the Candida Genome Database [45].

Gene name	Description	log_2_-fold change in EntV^68^ 4h	log_2_-fold change in EntV^68^ 24h	log_2_-fold change in 12mer 4h	log_2_-fold change in 12mer 24h
*C1_01510W*	Protein of unknown function; promoter bound by Bcr1, Efg1, Ndt80, and Rob1	1.27	1.26	3.2	2.21
*C6_04230W*	Uncharacterized	ND	ND	-1.56	ND
*C2_10650W*	Protein of unknown function; newly produced during adaptation to the serum	-1.08	-1.12	-2.76	-1.77
*RBR1*	GPI-anchored cell wall protein; expression repressed by Rim101 and activated by Nrg1	-1.26	ND	-4.55	-1.36
*AHP1*	Alkyl hydroperoxide reductase; Ssk1/Nrg1/Tup1/Ssn6/Hog1 regulated	-1.28	ND	-2.17	ND
*CR_03480W*	Uncharacterized	ND	ND	-1.17	-2.16

### Characterization of EntV-resistant mutants

The six genes highlighted in the RNA sequencing analysis (Tables 1 and S2) were further characterized. As the function of five of the six proteins is unknown, InterPro was used as a tool to provide functional analysis by classifying the proteins into families and predicting domains and important sites [45,46]. The only protein previously characterized is encoded by *AHP1*, which was described as an alkyl hydroperoxide reductase that enhances *C. albicans* adaptation to oxidative stress [47].

Three proteins, encoded by *C1_01510W, C6_04230W,* and *CR_03480W,* were predicted to be glycine-rich proteins associated with adhesion [48] and stress responses, notably in *Arabidopsis* [49]. Two proteins, encoded by *RBR1* and *C2_10650W*, contain synuclein domains, characterized by a lipid-binding motif with potential roles in vesicular trafficking [50].

**Fig 5:**
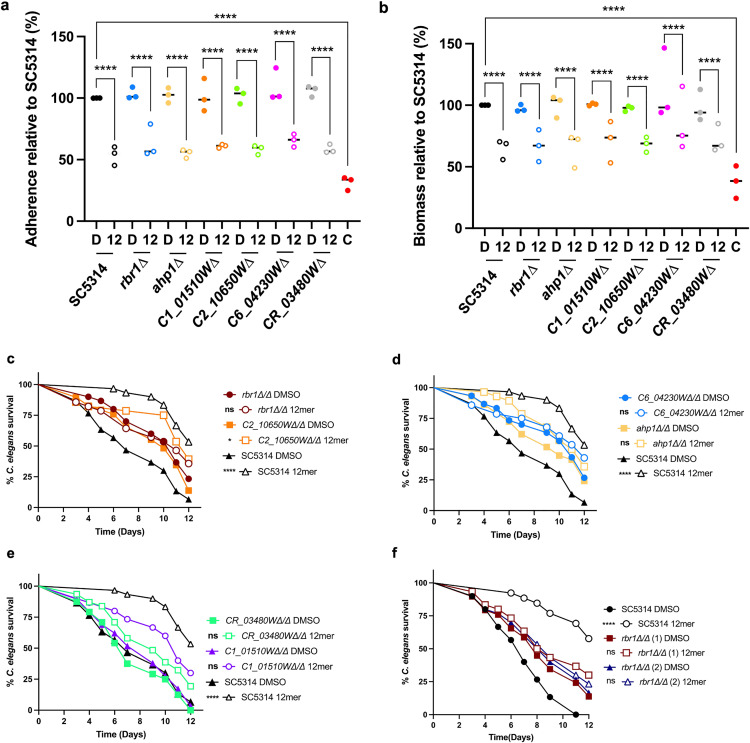
Characterization of EntV-resistant deletion mutants. (a) Relative adhesion in saliva medium at 37°C containing DMSO (D) or 12mer (12). (b) Relative biofilm biomass in saliva medium at 37°C for 48h. The rightmost column, “C” indicates the negative control, a biofilm-deficient *cph1∆ efg1∆* mutant. (c, d, e) Survival of *C. elegans* infected with *C. albicans* deletion mutants and exposed to 1nM 12mer. (f) Survival of *C. elegans* infected with two independent *C. albicans* *rbr1* deletion mutants. Statistical significance in comparison to the DMSO control group for each strain was determined using Mantel-Cox log-rank analysis. Median survival from biological replicates and the statistical comparisons of the mutant strains to the parent SC5314 are presented in S3 Table. *P < 0.05, **P < 0.01, ***P < 0.001, ****P < 0.0001.

To assess the involvement of these genes in susceptibility to EntV and various phenotypes related to virulence, deletion mutants of the ORFs were created in the *C. albicans* SC5314 background using CRISPR-Cas9 technology [51,52]. We assayed the potential for each of these mutants to adhere and form biofilms in vitro. Interestingly, the mutants were not found to be phenotypically different than the wild-type strain. After 48 hours of growth, none of the mutants exhibited any difference in adhesion and biofilm biomass compared to SC5314 in both artificial saliva medium (Fig 5a and 5b) and RPMI-1640 (S6c and S6d Fig). In accordance with this data, no alterations in the biofilm ultrastructure were noted using confocal microscopy (S6 Fig). However, the 12mer was able to still reduce adhesion and biofilm biomass in all the mutants in both types of media (Figs 5a, 5b, S6c, and S6c).

Next, the mutants were tested for resistance to the 12mer in the *C. elegans* model (Fig 5c-5e and S3 Table). We identified three general patterns in this assay. In the first, characterized by *rbr1∆, ahp1∆*, and *C6_04230W∆*, the mutants were insensitive to the peptide, but also attenuated in virulence. A fourth mutant, *C2_10650W∆*, also fit this pattern, but retained modest peptide sensitivity. Virulence of *C1_01510W∆* was normal, with only slight, statistically insignificant, inhibition by the 12mer. Lastly, *CR_03480W∆* also showed normal virulence, but was resistant to the peptide. To confirm these results, an independent clone of the *rbr1* deletion mutant was assessed for virulence in the worm assay, where it displayed similar attenuation of virulence and, more importantly, resistance to the 12mer (Fig 5f).

Furthermore, the deletion mutants were screened for additional phenotypes in a variety of assays, including growth, filamentation, adhesion, macrophage killing, stress resistance, and agar invasion, but no differences from the wild-type strain were observed (S4, S5, and S6 Figs). Interestingly, despite the resistance to the peptide noted in the *C. elegans* assays (Fig 5a-5d), no differences were observed in the binding/localization of the 12mer labeled with Alexa Fluor-647, compared to the wild type SC5314 (S7a Fig). These results were confirmed by comparing the focal distribution between SC5314 hyphae (Fig 1h) and the *rbr1* deletion mutant, which did not display significant variations in the fractions of cells stained with the 12mer peptide (S7b Fig).

A summary table of the mutants’ phenotypes is displayed in Table 2. Altogether, these data suggest that the genes identified by the RNA-sequencing analysis influence the mechanism of action of EntV, with phenotypes observed only in specific conditions.

**Table 2:** Summary of the phenotypes observed for the deletion mutants. The noted phenotypes are reported in comparison to the wild type (SC5314) and the extent was either similar (~), lower (-/---) or higher (+/+++).

Gene name	Filamentation	Virulence (*C. elegans*)	Virulence (macrophages)	Adhesion	Biofilm	Stress resistance	EntV resistance
*C1_01510W*	**~**	**~**	**~**	**~**	**~**	**~**	**+++**
*C6_04230W*	**~**	**---**	**~**	**~**	**~**	**~**	**+++**
*C2_10650W*	**~**	**---**	**~**	**~**	**~**	**~**	**+**
*RBR1*	**~**	**---**	**~**	**~**	**~**	**~**	**+++**
*AHP1*	**~**	**---**	**~**	**~**	**~**	**+**	**+++**
*CR_03480W*	**~**	**~**	**~**	**~**	**~**	**~**	**+++**

**Fig 6:**
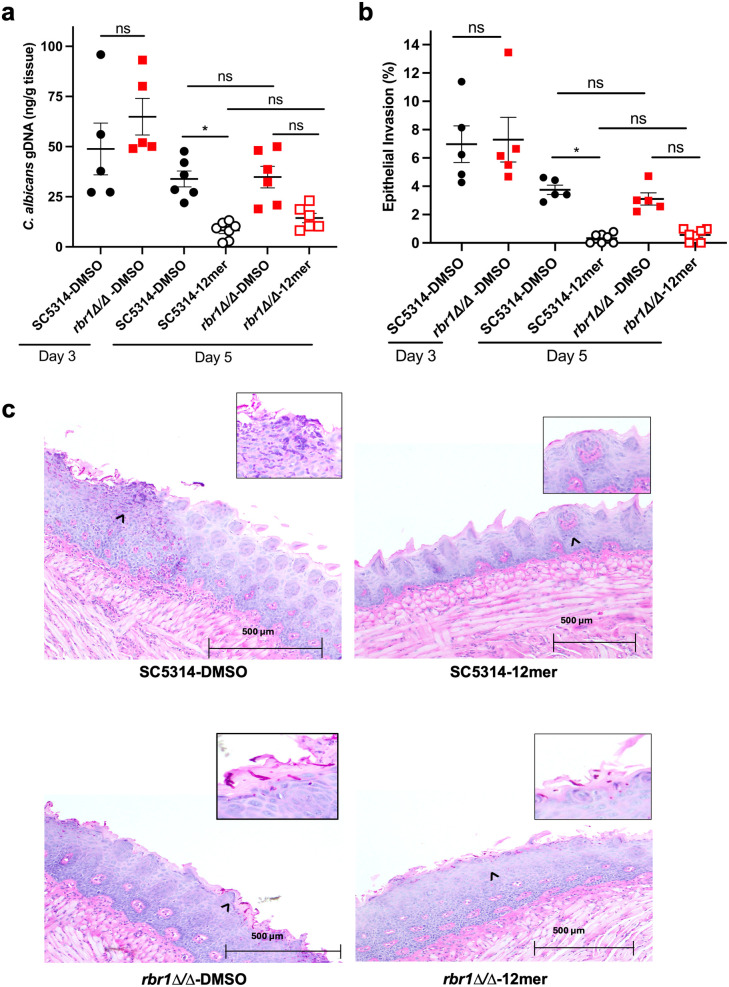
Efficacy of 12mer treatment in a mouse model of oropharyngeal candidiasis. (a) Fungal burden and (b) epithelial invasion of the tongue tissues from mice infected with *C. albicans* SC5314 and *rbr1∆* strains and treated with 100 nM of the 12mer peptide or the vehicle control (DMSO) given in the drinking water starting from day 3. *Candida* burden was measured using qPCR to quantify fungal DNA, and epithelial invasion was estimated by measuring the percentage of the tongue surface visibly disrupted, respectively. (c) Representative images used to score the hyphal invasion of the tongue tissue. *C. albicans* is represented by pink-stained cells in the areas that are magnified, indicated by the black arrows. The cells in the control SC5314 DMSO sample show that the hyphal morphotype of *C. albicans* is more prevalent than in the peptide-treated tissue samples. Statistical differences were compared by one-way ANOVA followed by Tukey’s multiple comparison test for all samples. *P < 0.05.

The development of resistance to EntV is a potential concern for developing the peptide as an antifungal agent. Based on previous work [30–32], the activity of the peptide in vivo has been widely described and characterized in an established model of murine oropharyngeal candidiasis [53]. For this reason, the *rbr1*∆ mutant was tested in this model in order to explore the therapeutic relevance of EntV resistance in vivo and how it affects the peptide treatment. Immunosuppressed outbred mice were infected sub-lingually with *C. albicans* SC5314 and *rbr1*∆ strains. After 3 days post-infection (d.p.i.), water containing 100nM of the 12mer peptide or DMSO was provided *ad libitum*. After 5 d.p.i., the mice were euthanized and the tongue tissues analyzed via qPCR and histology for fungal burden and epithelial invasion, respectively. As shown in Fig 6, both burden and invasion were significantly reduced in the group infected with the wild type and treated with the 12mer compared to the DMSO group (Fig 6a and 6b). For the *rbr1*-infected mice, a trend towards reduction in burden and invasion with the 12mer treatment was observed, suggesting that the resistance seen in the worm model might be partially mitigated in a mammalian model, but did not result in a statistically significant effect (Fig 6a and 6b).

In this OPC model, the *rbr1*∆ mutant was equivalent to the parent strain in both fungal burden and epithelial invasion (Fig 6a and 6b). However, some differences in morphology were observed by histology (Fig 6c). While hyphae were more abundant in mice infected with SC5314 and treated with DMSO, yeast and pseudo-hyphal morphotypes were present in animals infected with *rbr1*∆. As previously described [30], the 12mer treatment caused an arrest in filamentation and hyphal invasion in mice incubated with the wild-type strain (Fig 6c). These results indicate an impairment of the *rbr1* mutant in in vivo filamentation in the oropharyngeal environment, even in the absence of the peptide.

### The ESCRT machinery is involved in the EntV mechanism of action

**Fig 7:**
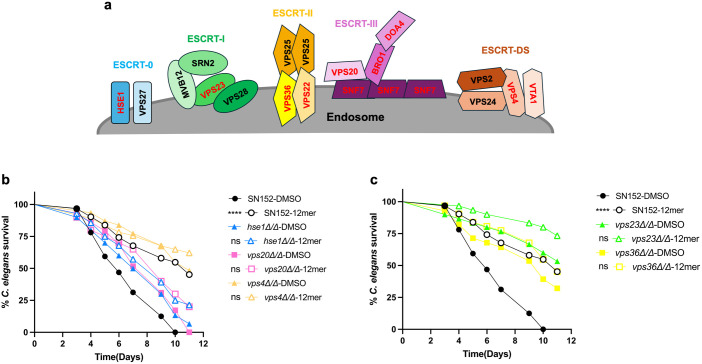
Screening of deletion mutants in the ESCRT pathway. (a) Schematic diagram of ESCRT machinery involved in EV production in eukaryotes. The deletion mutants tested for 12mer susceptibility are in red. (b, c) Survival of *C. elegans* infected with the *C. albicans* ESCRT component deletion mutants and treated with the 12mer peptide. Statistical significance in comparison to the DMSO control group for each strain was determined using Mantel-Cox log-rank analysis. The statistical comparisons of the mutant strains to the parent SN152 are presented in S3 Table. ****P < 0.0001.

The localization of EntV peptides and the involvement of possible vesicular trafficking proteins like Rbr1 led us to explore the EV-EntV link further. The biogenesis of EVs is dependent upon the packaging and sorting carried out by the Endosomal Sorting Complexes Required for Transport (ESCRT) pathway in eukaryotes [54] and abrogation of this pathway negatively affects biofilm and EV production in *C. albicans* [40]. We tested a panel of mutants defective in the subunits of the ESCRT pathway, known to reduce EV production, for their susceptibility to EntV-derived peptides (Fig 7a). Representative members of each complex of the ESCRT pathway were tested in the *C. elegans* survival assay to look for changes in 12mer susceptibility. Fig 7b and 7c show a subset of these data, which are summarized in S3 Table. In general, these mutants are less sensitive to inhibition by the peptides, but also significantly attenuated in virulence in the nematode model (Fig 7b and 7c and S3 Table). Overall, these results further corroborate the involvement of vesicular pathways in the EntV mechanism of action.

### EntV impacts EV number and size

**Fig 8:**
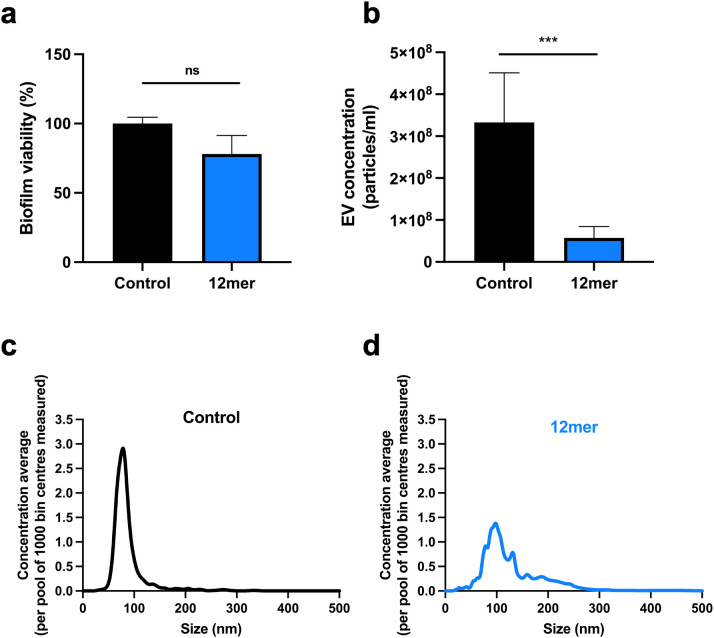
Analysis of EVs produced by *C. albicans* biofilms treated with EntV peptides. (a) Viability of *C. albicans* SC5314 biofilm following treatment with 1 µM of the 12mer peptide for 24 h in RPMI-1640 medium at 37°C. (b) Quantitative analysis of biofilm EVs assessed by nanoparticle tracking analysis (NTA). (c-d) Size distribution of *Candida* biofilm EVs determined by NTA. Statistical significance was determined using a T-test. *P < 0.05, **P < 0.01, ***P < 0.001.

We sought to test our hypothesis that the mechanism of action of EntV involves perturbation of EVs. EVs facilitate cell-to-cell communication and drive fungal biofilm development [55]. EntV is effective in disrupting biofilm formation and eradicating pre-formed biofilms in *C. albicans* [30,31]. Therefore, the effect of EntV on EVs from *Candida* biofilms was evaluated using nanoparticle tracking analysis (NTA), which can measure EV number and size. Vesicles were isolated from *C. albicans* SC5314 biofilms grown in RPMI-1640 medium for 24 hours at 37°C + /- 12mer (Fig 8). To ensure that the viability of the cells in the biofilms was not altered by presence of the peptide, a colorimetric tetrazolium reduction XTT assay was employed (Fig 8a). No differences in metabolic activity were detected when EntV peptide was added (Fig 8a), confirming that the peptide does not affect viability. Conversely, a six-fold reduction in EV production was observed after 24 hours incubation with the 12mer (Fig 8b). Furthermore, EVs from biofilms treated with the 12mer were far more heterogenous in their size, with a decrease in the output of vesicles sized in the 80–100 nm range and an increase in larger EVs (>100 nm) (Fig 8c and 8d). The larger vesicles and greater heterogeneity are typical of yeast-form cells and consistent with observations that EntV peptides reduce hyphal formation in biofilms.

## Discussion

The antifungal activity of EntV was previously isolated to a 12-amino acid peptide that was protective in nematode and rodent models of infection with efficacy against multiple fungal pathogens. As the work reported here was ongoing, we identified a 10-amino acid variant that retains full activity (P4-L) [32]. Here, we further characterized the non-fungicidal, non-fungistatic mechanism of action. We discovered that the 12mer peptide binds to structures associated with the cell envelope and colocalizes with EV-specific dyes, consistent with EntV targeting EVs. Transcriptomics and genetics reinforce an EV-mediated mechanism of action, as treatment with the peptide reduces EV production and increases the heterogeneity of these particles. Because EVs have been implicated in fungal virulence and host-pathogen interactions [56], an antifungal peptide that targets these vesicles represents a compelling mechanism of action.

A fluorescently labelled 12mer binds to the surface of *C. albicans* (yeast and hyphal morphologies) as well as other *Candida* species (i.e., *C. parapsilosis* and *C. auris*) in a punctate and dynamic fashion as visualized via confocal microscopy (Fig 1a-1f and S1 and S2 Movies). This staining is similar to that of the full-length EntV detected by indirect immunofluorescence. Moreover, the peptide appeared to preferentially bind hyphal cells compared to yeast (Fig 1g-1i). These data also suggest that the target of the peptide is more abundant in hyphae than in yeast. The localization of the 12mer to extracellular vesicles was demonstrated using two lipophilic dyes known to recognize EVs, and resembles the staining pattern previously described as exosomes in *Cryptococcus* [57] (Fig 3). While internalization of some forms of EntV were reported in another study [58], we observed no internalization or direct binding with intracellular components at the lower concentrations used in our work (Fig 1l and 1m and S1, S2 and S3 Movies). Overall, our work supports a model in which EntV localizes to a target associated with EVs, which is more abundantly expressed on EVs arising from *C. albicans* hyphae.

Interestingly, the antifungal activity of the 12mer does not require a specific stoichiometry, as demonstrated by the variability in peptide clusters detected via STORM (Fig 2f). Combined with the lack of fungicidal activity and hydrophobic nature, this highlights a unique mechanism of action compared to the traditional pore-forming activity of other antifungal peptides [33]. Moreover, we showed previously that there is considerable sequence flexibility in the 12mer, in which no single amino acid is essential for activity. Earlier data suggested that the C-terminal cysteine was the exception, as peptides lacking this residue were less active [31]. Here, we demonstrate that the role of the cysteine is more complicated than previously appreciated. Replacement of the cysteine with the structurally similar serine significantly reduced both activity and surface binding (Fig 2c and 2e). Surprisingly, substitution with the non-polar and non-reactive alanine had little effect on either binding or activity (Fig 2b and 2e). In the native 68aa protein, formation of a disulfide between this cysteine and one near the amino terminus is required for activity; this is possibly a structural requirement to expose the helix that includes the 12mer. Our mass-spectrometry data suggest that the WT 12mer peptide is mostly a dimer mediated by the cysteine residues. Both of these observations indicate that the thiol-reactivity of the cysteine is not essential to the activity of the peptide. The reduced activity of the serine substitution, relative to the alanine, might indicate that polarity or reactivity is detrimental, perhaps by sequestering the peptide in the hydrophilic cell wall via covalent or hydrogen bonding. The exact requirements for this C-terminal residue will require more investigation.

The ESCRT machinery is pivotal in several cellular processes, including EV biogenesis and biofilm matrix assembly [40]. *C. albicans* mutants for components of this pathway displayed altered levels of susceptibility to EntV in a *C. elegans* survival assay (Fig 7), supporting the hypothesis that alterations in EV production and/or function are involved in the peptide’s mechanism of action. A nanoparticle tracking analysis of EVs from biofilms treated with the 12mer demonstrated the impact of EntV on EV production (Fig 8). A reduction in the total vesicular output as well as a shift towards the secretion of larger vesicles (>100 nm) was detected upon incubation with the 12mer (Fig 8b-8d). Hyphal and biofilm EVs are smaller and more homogenous than the ones secreted by yeast and planktonic cells [40,59]. Since EntV has a detrimental effect on filamentation, adhesion, and biofilm formation [30,31], the production of larger-sized vesicles could be an effect of the 12mer-induced shift towards the planktonic state for the treated cells. Another novel antifungal compound, turbinmicin, was reported to target the fungal EV pathway [60] by targeting Sec14p, which is important for the vesicular trafficking pathway. Turbinmicin displayed high efficacy in infection models against multiple fungal pathogens [60,61]. Despite this, turbinmicin is structurally unrelated to EntV and is fungicidal [30]. Nevertheless, this strengthens the connection between EVs and fungal virulence.

We performed a transcriptome analysis of cells incubated with EntV peptides, discovering that a minimal set of genes of unknown function was differentially expressed between the control and the EntV-treated cells (Fig 4c). While mutants of these genes did not show phenotypes in a variety of host-relevant assays (Figs 5a, 5b, S4, S5, and S6), the strains were generally less susceptible to the 12mer in the worm model (Fig 5a-c). Interestingly, all the resistant strains tested (including the ESCRT mutants) still bound the peptide in the same punctate pattern observed in the wild type strain (S7 Fig). We can therefore speculate that the development of resistance to the peptide does not necessarily involve a change in the binding to the target, but rather an alteration of the molecular pathway(s) involved in EV formation, release, or cargo components. This is consistent with our data indicating that EV production and size are altered after EntV treatment. Because many of these mutants are impaired in virulence, developing resistance to the peptide may carry fitness costs and not be as consequential during infections (Figs 5c-5f, 7b, and 7c). In a murine OPC model, the *rbr1∆* mutant still trended towards susceptibility to the peptide and was seen to be less hyphal and less invasive (Fig 6). We observe some discrepancies between the effect of these mutants and their susceptibilities to EntV between in vitro, nematode, and mouse models. *C. albicans* virulence is multifactorial and behaviors important in one context may not necessarily be important in others, so we do not find these differences surprising and may be able to use them to parse out the relative contributions of EVs in these different models. However, further work is needed to identify the specific target of EntV and understand its role in the peptide’s mechanism of action.

The presence of virulence factors, such as Ece1p [62] and Sap4, -5, and -6 [63], which are reportedly essential for systemic infections and highly immunogenic, in EVs has been described [40,59]. Additionally, EVs are also reported to have immunomodulatory effects [64,65]. The decreased production accompanied by the shift in size of EVs occurring during 12mer treatment could be associated with a change in protein cargo of the vesicles that affects key virulence traits. These changes in EV cargo could also influence the host immune response and contribute to the treatment efficacy of EntV observed in infection models. Further studies will be needed to better define the immune system’s role in the mechanism of action of this peptide in vivo.

In conclusion, this work provides insight into the novel mechanism of action of EntV-derived peptides, showcasing their potential as broad-spectrum antifungals. The identification of EVs as the primary target of EntV, like turbinmicin [61], provides a strong rationale for considering EVs as a druggable target for developing future antifungal compounds.

## Materials and methods

### Ethics statement

**All animal procedures were conducted in accordance with protocols approved by the Animal Welfare Committee of the University of Texas Health Science Center at Houston.**

### Strains and media

The strains used for this work are listed in S1 Table. Fungal strains were grown and propagated in YPD medium (1% yeast extract, 2% peptone, 2% dextrose). For induction of hyphae, cells were grown in RPMI-1640 medium at 37°C for 3 h without shaking.

For phenotypic assessment of *C. albicans* mutants, cells were grown on YPD agar plates containing either 10 mM caffeine, 300 mM lithium chloride (LiCl), or 0.05% sodium dodecyl sulphate (SDS) at 30°C. For oxidative stress assays, cells were grown on YPD medium supplemented with 10 mM hydrogen peroxide for 24 h at 30°C.

Deletion mutants were generated by the CRISPR/Cas9 SAT-Flipper method [66,67]. Briefly, the open reading frames were replaced with an *FLP1-SAT1* cassette from the pSFS2 plasmid [68]. Mutants were verified via PCR, and the *FLP1-SAT1* cassette was flipped out via maltose-induced activation of the flippase.

### Immunofluorescence

Fungal cells were grown in YPD at 30°C with shaking overnight. The cells were then centrifuged at 870xg for 5 min and washed twice with phosphate-buffered saline (PBS). For induction of *C. albicans* hyphae, cells were incubated in IBIDI chamber slides in RPMI-1640 medium at 37°C for 3 h without shaking. Samples were subsequently inactivated by formaldehyde, as previously described [69]. For fluorophore labeling of the peptides, copper-free click chemistry [70] was used, between a modified lysine at the N-terminus of the 12mer sequence containing an azide group on the side chain and the strained dicyclobutenyl (sDIBO) alkyne on the fluorophore. The labeled peptides were then purified via reverse-phase high-pressure liquid chromatography. Briefly, cells were washed in PBS and incubated for 1 h at room temperature with 4% formaldehyde solution in 0.1 M PHEM buffer (120 mM PIPES, 50 mM HEPES, 4 mM MgCl_2_*6H_2_O, 20 mM EGTA, pH 6.9), before overnight storage at 4°C. They were washed with PBS three times before use. The incubation with the 12mer peptides was performed for 2 h at room temperature with shaking (30rpm) using 1 µM fluorescently labelled peptides. The cells were washed three times with PBS and stained with 50 µg/mL calcofluor white (Fluorescent Brightener 28, Sigma–Aldrich Co.), 125 µg/mL concanavalin A-Alexa Fluor-647, 0.2 µM DiIC_18_ [5]-DS (D12730, Thermo Scientific), 0.5µM Vybrant Dil Cell-Labeling Solution (V22885, Thermo Scientific). For immunolabeling of EntV^68^, samples were incubated with a rat α-EntV polyclonal antibody (1:250) for 1 h at room temperature, washed three times in PBS and then labeled with an α-rat-Alexa Fluor-594 secondary antibody diluted 1:1000 for 1h at room temperature. Post-incubation, all samples were washed three times before imaging. All the images were acquired as Z-stacks and were obtained using the 100x objective of an Olympus IX-83 spinning-disk confocal microscope. For 3D rendering, the Z-stack images were interpreted using BiofilmQ [38]. Denoising and declumping were performed on a scale of 25 voxels and by 8 voxels, respectively. The rendering was visualized in Paraview [71], setting the opacity of the fungal cells from 1.0 to 0.3.

### Quantification of 12mer foci

To measure the differential distribution of 12mer binding between yeast and hyphae, *C. albicans* SC5314 cells were grown as previously described. After formaldehyde inactivation, samples were incubated with 0.5µM 12mer-Alexa Fluor-488 for 2 h at room temperature with gentle shaking (30rpm), then stained with 50 µg/mL calcofluor white for 10 min at room temperature. After three washes in PBS, the images were obtained at 100x with an Olympus IX-83 spinning-disk confocal microscope, with Z-stacks of 0.2 µm generated. Three biological replicates were performed, with at least 10 images collected for three technical replicates each. For the calculation, one hyphal segment was defined as the part of the cell between two septa.

### STORM microscopy

The samples were prepared for STochastic Optical Reconstruction Microscopy (STORM) following the same protocol used for fluorescence confocal microscopy. Single-molecule localization and clusters were determined using density-based spatial clustering of applications with noise (DBSCAN) tool in the NIS-Elements software package. The imaging experiments were conducted on an N-STORM Eclipse Ti2 inverted microscope (Nikon Instruments). STORM images were collected in a 512 x 512-pixel region of interest using a CFI Apochromat TIRF 100x (NA 1.49) oil objective (Nikon Instruments) and a C11440-22CU ORCA-flash sCMOS 4.0 V2 camera (Hamamatsu). Images were acquired sequentially, 500 frames per filter channel, at a 300-ms duration.

### Matrix-assisted laser desorption/ionization time-of-flight (MALDI-TOF)

For the dimerization analysis, the peptides were analyzed using a Bruker Autoflex Speed MALDI operated in positive ion and reflectron mode. Samples were mixed 1:1 (v/v) with a saturated solution of α-Cyano-4-hydroxycinnamic acid (CHCA) matrix, prepared in 70% acetonitrile, 30% water, and 0.1% trifluoroacetic acid. After mixing, ~ 1 µL of the sample was spotted onto the MALDI plate and allowed to dry at room temperature before MALDI-MS analysis.

### Transcriptome analysis

*C. albicans* transcriptional response to EntV peptides was analyzed by growing the cells in the same settings of the biofilm assays, following our published protocol [32]. Briefly, cells were grown in YPD for 18 hrs at 30°C, washed in PBS, and adjusted to a concentration of 10^7^ cells/mL in PBS with 100nM peptide. The cell suspension was incubated for 1 hr at 30°C at 200 rpm. Then, the cell suspension was aliquoted into 6-well tissue culture-treated polystyrene plates (Falcon) and incubated at 37°C for 1 hr at 200 rpm. The PBS was then removed and replaced with artificial saliva medium [30,43] containing 100 nM peptide. The plates were incubated at 37°C for 4, 24, and 48 hrs. The RNA isolation was performed using the RiboPure RNA Purification Kit (Ambion). The sample quality was assessed using the Agilent 2100 Bioanalyzer (Agilent Technologies) and then used to prepare the cDNA libraries by employing the Illumina TruSeq sample preparation protocol. The sequencing was performed using a NovaSeq sequencing platform (Illumina). Adapter sequences were trimmed using trim_galore v.0.6.6 (cutadapt v. 2.5). Sequencing reads were then aligned to the *C. albicans* reference genome sequence [45] using STAR v.2.5.2a aligner [72]. All downstream analyses were conducted using the R programming language for statistical computing and visualization. Processing of gene expression data, including normalization, principal component analyses, analysis of similarities across samples, and differential gene expression testing were conducted using DESeq2 [73].

### *C. albicans* adhesion and biofilm assays

To test adhesion and biofilm formation of *C. albicans* deletion mutants, the methodology used in [31] was carried out. Briefly, *C. albicans* strains were grown in YPD broth overnight at 30°C with shaking and then subcultured for 4 h in the same conditions. After two washes in PBS, 1 × 10^7^ cells/ml were collected and incubated in PBS for 1 h at 30°C with shaking. Then 100µL of cell suspension was added to 100µL of saliva medium [30] and aliquoted in a 96-well plate for 90 min (adhesion) or 24 and 48 h (biofilm) at 37°C without shaking. At each time point, the supernatant was removed and the cell pellet stained with 0.08% crystal violet solution (Sigma) for 20 min. The crystal violet was then removed, 200 µl of 200 proof ethanol was added for 20 min, and the OD_595_ of the supernatants was measured using a Synergy H1 plate reader (BioTek) with Gen5 version 3.08 software (BioTek).

### Biofilm peptide susceptibility assay

Peptide susceptibility of *Candida* biofilms was evaluated using 96-well flat-bottom polystyrene plates. Overnight yeast cultures grown in YPD at 30°C were used to prepare fungal cell inocula at a concentration of 10⁶ cells/ml. Cell counts were determined with an automated Countess II cell counter (Invitrogen), and the cells were diluted in RPMI-MOPS accordingly. Then, 100 μl of yeast suspension was seeded into each well. Biofilm formation proceeded for 6 hours. After this period, nonadherent cells were removed by washing the wells twice with phosphate-buffered saline (PBS, pH 7.2). The tested peptides at a concentration of 1µM, along with fresh RPMI medium, were added to the wells, and biofilms were allowed to develop for 24 hours. Following incubation, the supernatants were removed, filter sterilized, and used for subsequent extracellular vesicle analysis. To assess biofilm viability, the colorimetric tetrazolium reduction XTT assay was employed. The percent reduction in biofilm growth was calculated based on the decrease in absorbance compared to untreated controls. For the assay, XTT (2,3-bis[2-methoxy-4-nitro-5-sulfophenyl]-2H-tetrazolium-5-carboxanilide inner salt) was freshly prepared at a concentration of 0.75 mg/mL, and phenazine methosulfate (2 mM) was added as an electron acceptor to enhance the reduction process. Absorbance was measured at 492 nm using an automated Cytation 5 imaging plate reader (BioTek).

### Extracellular vesicle analyses

Exosome quantification was performed using nanoparticle tracking analysis. First, extracellular vesicle samples were diluted in PBS to a final volume of 1 ml and pretested with a NanoSight NS300 system (Malvern Instruments) to achieve an optimal concentration of 30–100 particles per frame. The camera settings were adjusted—specifically, the camera level was set to 16 and the gain to 2—to ensure clear visualization of nanoparticles without causing signal saturation. Each sample was analyzed by capturing five 1-minute videos, with a 5-second delay between sample introduction and the start of the first video. During detection threshold analysis, counts were controlled so that only 10–100 red crosses and no more than 5–7 blue crosses were visible. Data processing was carried out using NanoSight Software NTA 3.4 (Build 3.4.003), and a minimum of 1,000 events were tracked per sample to reduce the impact of data skewing from individual large particles.

### *C. elegans* infection assay

To prepare the infection plates, fungal strains were grown in BHI broth overnight at 30°C with agitation. 500 μL of the culture was plated onto BHI solid medium containing gentamycin (10 μg/mL) and grown for 24 hrs at 37°C. Synchronized L4 *C. elegans* nematodes were then washed off the OP50 plates in 2 mL sterile M9 buffer and washed once, centrifuging at 800 rpm for 30 seconds to collect the nematodes. Nematodes were infected by placing them on the fungal lawn for 4 hrs at 25°C. Following this exposure, they were washed off the plate and washed four times with 2 mL of sterile M9. The nematodes were then pipetted (~30 per well with two wells per condition for a total of ~60 worms assayed) into six-well plates with 2 mL of liquid medium (20% BHI broth and 80% M9) containing 1 nM of peptide. A DMSO untreated control was used with each experiment. Plates were incubated at 25°C, and worm death was scored daily. Kaplan–Meier survival curves were generated, and Mantel-Cox log-rank analysis was used to compare curves. The median survival and comparison values for all *C. elegans* survival experiments and their replicates can be found in S3 Table.

### Murine oropharyngeal candidiasis model

The efficacy of EntV peptides was tested in an oropharyngeal candidiasis model [30–32,53]. ICR mice, 7–9 weeks old and approximately 18–20g in weight, were immunosuppressed by injecting 225 mg/kg cortisone acetate subcutaneously 1 day before inoculation and subsequently on days 1 and 3 post-inoculation. To prepare the fungal inoculum, 1mL of the *C. albicans* strain’s overnight culture, grown at 30°C in YPD broth, was washed twice in PBS before resuspension in sterile Hanks’ Balanced Salt Solution (HBSS) at a concentration of 1 × 10^6^ cells/mL. Calcium alginate swabs were soaked in this inoculum for 10 min before inoculation. Mice were anesthetized using a mix of ketamine (100 mg/kg), acepromazine (2 mg/kg), and xylazine (10 mg/kg) and placed on pre-warmed isothermal pads. The swabs were placed sublingually for 75 min. Mice were given additional doses of ketamine (50 mg/kg) as necessary. At day 3 post-inoculation, mice were given drinking water containing the 0.1 µM 12mer peptide or vehicle control (0.01% DMSO) *ad libitum*. Mice were euthanized on day 5 after inoculation. The tongues were then extracted and cut in half laterally for tissue histology and measurement of fungal burden.

For tissue histology, half the tissue sample was placed in 10% zinc-buffered formalin overnight and stored in 80% ethanol before being embedded in paraffin. For each tissue, 5-µm sections were prepared using a Leica microtome and stained using Periodic Acid-Schiff (PAS) stain. All sections were scanned using a light microscope at 40x magnification for histopathological analysis. The other half of the tongue was homogenized for assessment of fungal burden via qPCR. DNA was extracted using the Yeast DNA Extraction Kit (Thermo Scientific), and qPCR was used for amplifying a 269-bp fragment of the internal transcribed spacer 2 (ITS2) between the 5.8S and 28S ribosomal RNA genes of *C. albicans*. The qPCR was performed with the FastStart Universal SYBR Green master mix with the ROX kit (Roche) using a CFX96 Real-Time System with a C1000 Touch thermal cycler (BioRad). To screen for contamination and background fluorescence during qPCR amplification, no-template controls were used.

### Quantification and statistical analysis

GraphPad Prism 9.0 was used for all data analysis. For the oropharyngeal candidiasis measurements, means of the experimental conditions were calculated and compared to other conditions as indicated in the individual panels. Lines with error bars indicate the mean and the standard error of the mean (SEM). Significance was determined using one-way ANOVA followed by Tukey’s multiple comparison test. For the *C. elegans* survival assays, Mantel–Cox log-rank analysis was used to compare survival curves. The median survival and comparison values for all *C. elegans* survival experiments and their replicates can be found in S3 Table. For all statistical tests, P values <0.05 were considered statistically significant and asterisks in the figure panels indicate the levels of significance as follows: *P < 0.05, **P < 0.01, ***P < 0.001, ****P < 0.0001.

## Supporting information

S1 Fig Negative controls for EntV^68^ staining. Fluorescence microscopy of *C. albicans* SC5314 labeled with: **(a)** 1 µM EntV^68^ and an Alexa Fluor-594 secondary antibody; **(b)** anti-EntV^68^ and an Alexa Fluor-594 secondary antibody; **(c)** an Alexa Fluor-594 secondary antibody. The plasma membrane was labeled with Pma1-GFP. The images were acquired using an Olympus IX-83 microscope as described in the Materials and Methods section.
(TIF)

S2 Fig Efficacy of fluorescently labeled peptides in the worm model. Survival of C. elegans infected with *C. albicans* SC5314 and exposed to 1nM 12mer (red), 12mer-Alexa Fluor-647 (blue) or 12mer-Alexa Fluor-488 (purple). AF647 = Alexa Fluor-647; AF488 = Alexa Fluor-488. Statistical significance in comparison to the DMSO control group was determined using Mantel-Cox log rank analysis. ****P < 0.0001.
(TIF)

S3 Fig Dimerization analysis of 12mer peptides via MALDI-TOF mass spectrometry. The wild type 12mer **(a)** displayed dimer formation, identified by the peak 2535.6 m/z. The cysteine to alanine **(b)** and to serine **(c)** mutants did not show the presence of any dimers, with just the peaks of the single peptides at 1247.7 and 1263.7 m/z, respectively. The peptides were analyzed using a Bruker Autoflex Speed MALDI operated in positive ion and reflectron mode.
(TIF)

S4 Fig Growth and filamentation of *C. albicans* mutants. *C. albicans* deletion mutants were generated via CRISPR-Cas9 technology and assessed for growth rates in YPD medium at 30°C **(a);** hyphal formation on YPD agar medium at 37°C **(b)** and on 10% fetal bovine serum at 37°C **(c)**.
(TIF)

S5 Fig Phenotypic assessment of *C. albicans* mutants. *C. albicans* deletion mutants were tested for: growth on YPD agar plates containing either 10 mM caffeine, 300 mM lithium chloride (LiCl) or 0.05% sodium dodecyl sulphate (SDS) at 30°C **(a);** growth on 10 mM hydrogen peroxide (H_2_O_2_) on YPD medium at 30°C **(b)**; killing of J774A.1 macrophages in RPMI-1460 medium at 37°C + 5% CO_2_ **(c)**; agar invasion on 10% fetal bovine serum **(d)**. Statistical differences were compared by one-way ANOVA followed by Tukey’s multiple comparison test for all samples. *P < 0.05, ****p < 0.0001.
(TIF)

S6 Fig Biofilm assessment of *C. albicans* mutants. **(a)** The biofilm architecture of *C. albicans* deletion mutants was assessed by confocal microscopy on cells grown in RPMI-1460 medium at 37°C for 48 h. The biofilms were stained with calcofluor white (blue). **(b)** Relative adhesion in RPMI-1460 medium at 37°C. **(c)** Relative biofilm biomass in RPMI-1460 medium at 37°C for 48h. Statistical significance in comparison to the DMSO control group for each strain was determined using one-way ANOVA followed by Tukey’s multiple comparison test for all samples. *P < 0.05, ****P < 0.0001.
(TIF)

S7 Fig *C. albicans* mutants stained colocalizes with 12mer peptide. **(a)** Fluorescence microscopy of *C. albicans* hyphae of deletion mutants labeled with 1 µM 12mer-Alexa Fluor-647 (purple) and stained with calcofluor white (blue). **(b)** Foci distribution of *C. albicans* rbr1∆/∆ hyphae stained with the 12aa-Alexa Fluor-488. Statistical differences were compared to *C. albicans* SC5314 hyphae by one-way ANOVA followed by Tukey’s multiple comparison test for all samples.
(TIF)

S1 Table Strains used in this work. (XLSX)

S2 Table Normalized RNA-seq data from EntV and 12mer-treated samples. Tabs show differential gene expression, as described in the Materials and Methods, for the tested conditions.
(XLSX)

S3 Table Statistical summary of *C. elegans* infection experiments. These extended data support the *C. elegans* killing assays shown in Figs 2e, 5c-5f, and 7b-7c.
(XLSX)

S1 Movie EntV binding is punctate and dynamic. Time-lapse microscopy of *C. albicans* SC5314 hyphae expressing *adh1*-mCherry and labeled with 1µM EntV^68^-FITC. The videos were acquired using an Olympus IX-83 microscope as described in the Materials and Methods section.
(MP4)

S2 Movie EntV binding is punctate and dynamic. Time-lapse microscopy of *C. albicans* SC5314 yeast expressing *adh1*-mCherry and labeled with 1µM EntV^68^-FITC. The videos were acquired using an Olympus IX-83 microscope as described in the Materials and Methods section.
(MP4)

S3 Movie Cell surface binding of the 12mer peptide. BiofilmQ 3D of a biofilm stained with 1µM 12aa-Alexa Fluor-647 peptide (l, pseudocolored in red) superimposed on the *C. albicans* hyphae (m, pseudocolored in light blue) highlights the binding of the peptide throughout the biofilm. Scale bars represent 20 µm in the x, y, and z directions. The video was acquired using an Olympus IX-83 microscope as described in the Materials and Methods section.
(TIF)
