# Supplementary figures and images for: The antifungal mechanism of EntV-derived peptides is associated with a reduction in extracellular vesicle release
Source: PLoS Pathog. 2025 Sep 22;21(9):e1013519. doi: 10.1371/journal.ppat.1013519 (PMC12510648; doi:10.1371/journal.ppat.1013519)

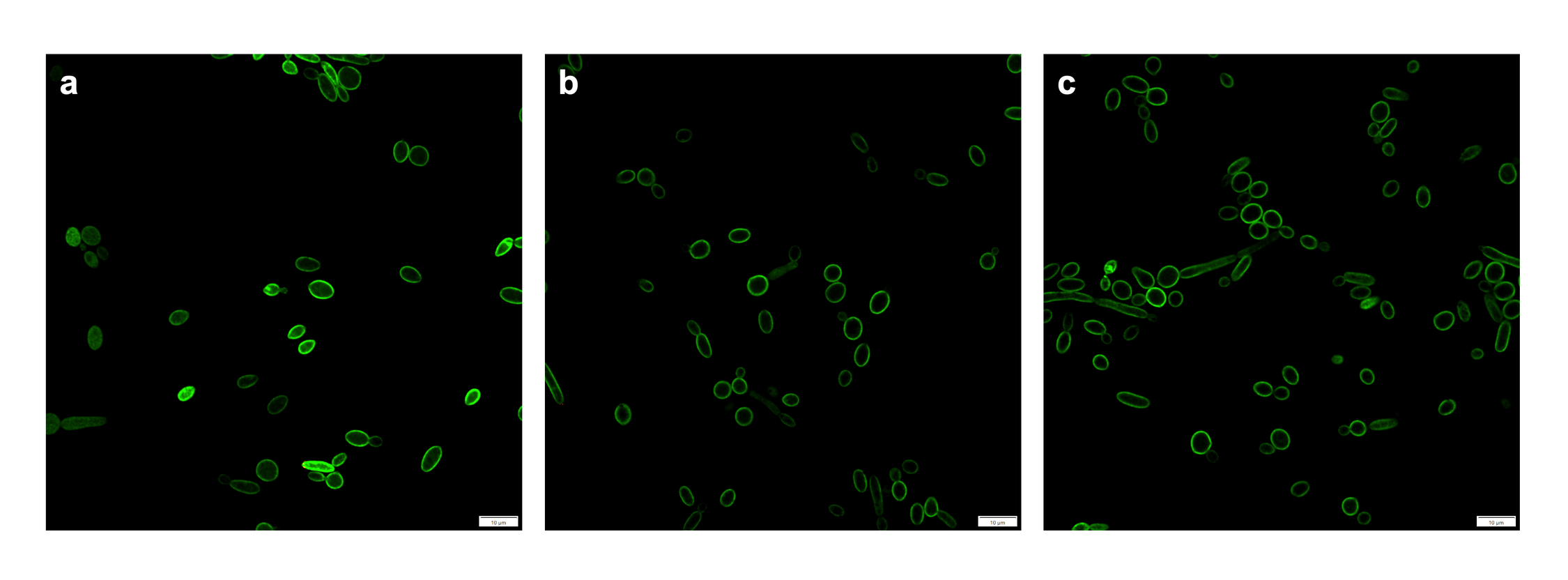

Supplement: S1 Fig — Fluorescence microscopy of C. albicans SC5314 labeled with: (a) 1 µM EntV68 and an Alexa Fluor-594 secondary antibody; (b) anti-EntV68 and an Alexa Fluor-594 secondary antibody; (c) an Alexa Fluor-594 secondary antibody. The plasma membrane was labeled with Pma1-GFP. The images were acquired using an Olympus IX-83 microscope as described in the Materials and Methods section. (TIF) [file ppat.1013519.s001.tif]

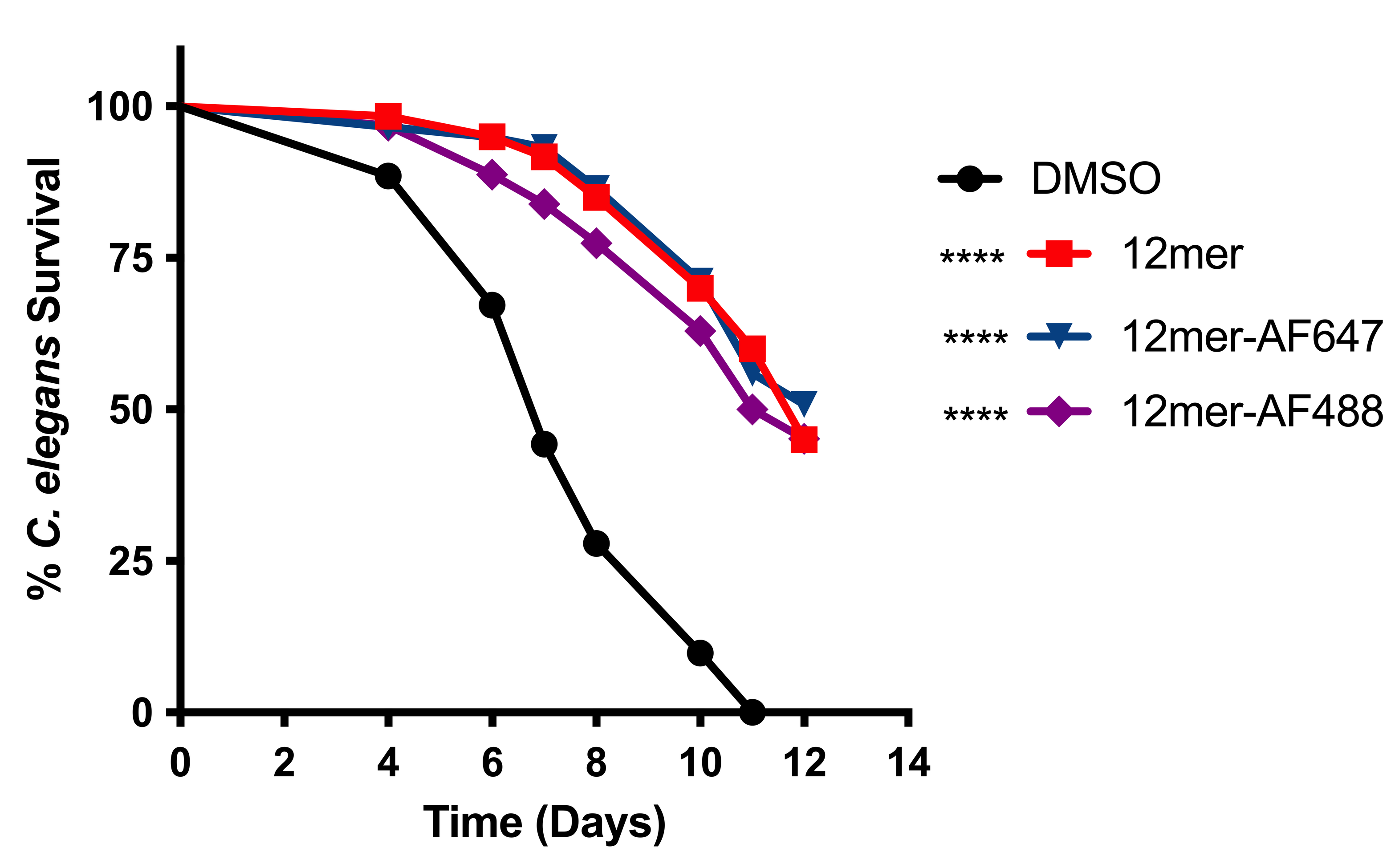

Supplement: S2 Fig — Survival of C. elegans infected with C. albicans SC5314 and exposed to 1nM 12mer (red), 12mer-Alexa Fluor-647 (blue) or 12mer-Alexa Fluor-488 (purple). AF647 = Alexa Fluor-647; AF488 = Alexa Fluor-488. Statistical significance in comparison to the DMSO control group was determined using Mantel-Cox log rank analysis. ****P < 0.0001. (TIF) [file ppat.1013519.s002.tif]

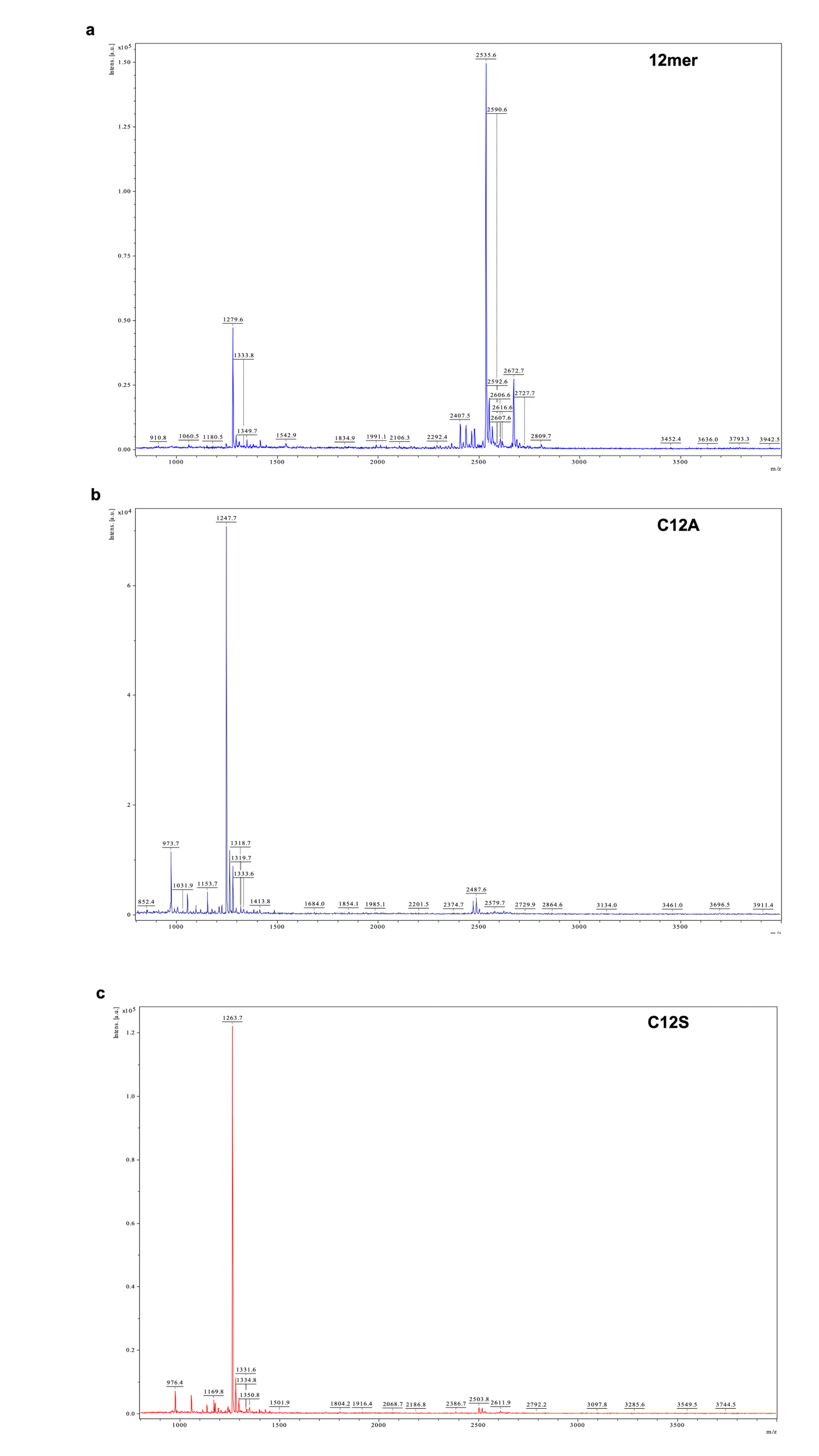

Supplement: S3 Fig — The wild type 12mer (a) displayed dimer formation, identified by the peak 2535.6 m/z. The cysteine to alanine (b) and to serine (c) mutants did not show the presence of any dimers, with just the peaks of the single peptides at 1247.7 and 1263.7 m/z, respectively. The peptides were analyzed using a Bruker Autoflex Speed MALDI operated in positive ion and reflectron mode. (TIF) [file ppat.1013519.s003.tif]

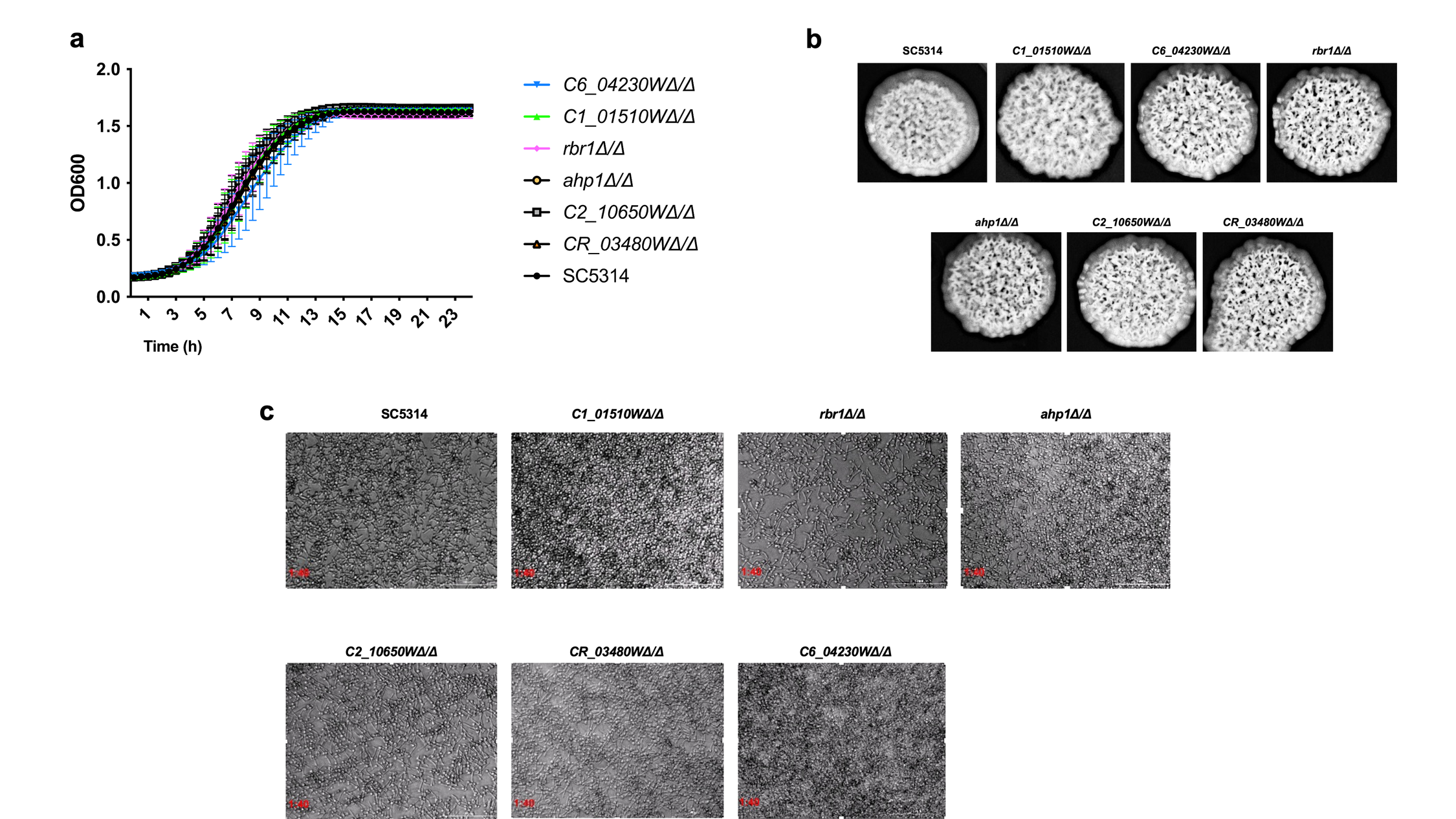

Supplement: S4 Fig — C. albicans deletion mutants were generated via CRISPR-Cas9 technology and assessed for growth rates in YPD medium at 30°C (a); hyphal formation on YPD agar medium at 37°C (b) and on 10% fetal bovine serum at 37°C (c). (TIF) [file ppat.1013519.s004.tif]

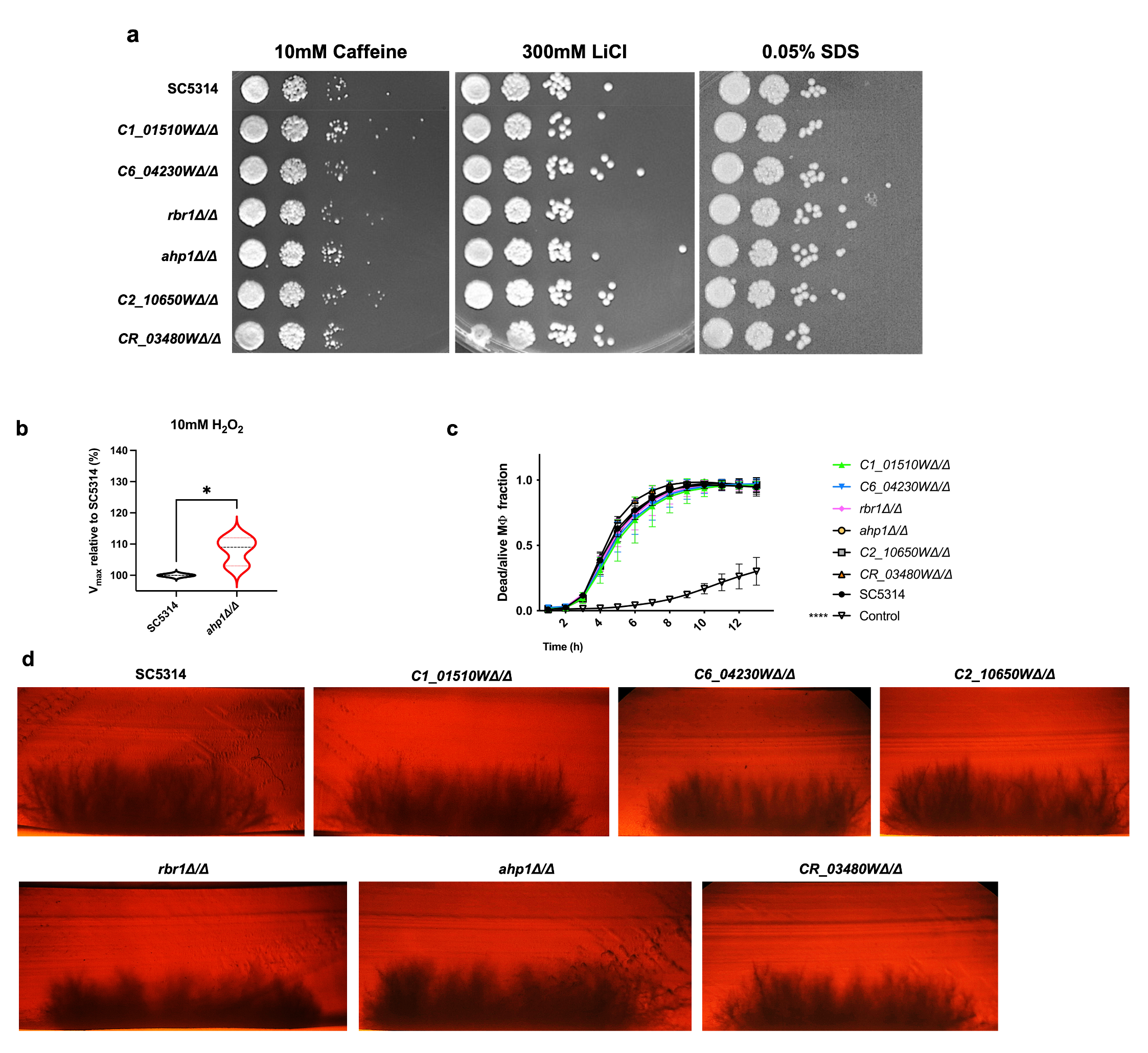

Supplement: S5 Fig — C. albicans deletion mutants were tested for: growth on YPD agar plates containing either 10 mM caffeine, 300 mM lithium chloride (LiCl) or 0.05% sodium dodecyl sulphate (SDS) at 30°C (a); growth on 10 mM hydrogen peroxide (H2O2) on YPD medium at 30°C (b); killing of J774A.1 macrophages in RPMI-1460 medium at 37°C + 5% CO2 (c); agar invasion on 10% fetal bovine serum (d). Statistical differences were compared by one-way ANOVA followed by Tukey’s multiple comparison test for all samples. *P < 0.05, ****p < 0.0001. (TIF) [file ppat.1013519.s005.tif]

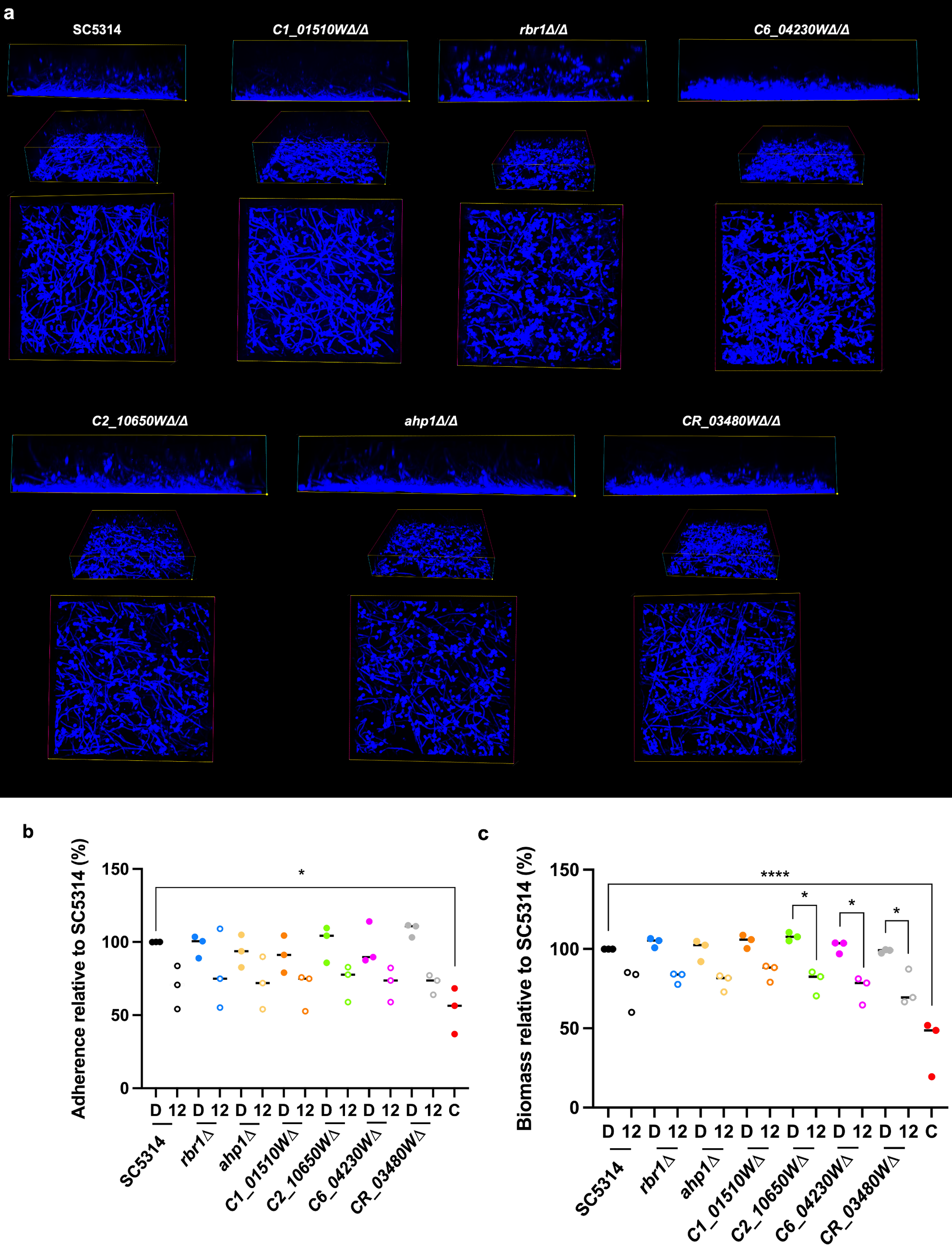

Supplement: S6 Fig — (a) The biofilm architecture of C. albicans deletion mutants was assessed by confocal microscopy on cells grown in RPMI-1460 medium at 37°C for 48 h. The biofilms were stained with calcofluor white (blue). (b) Relative adhesion in RPMI-1460 medium at 37°C. (c) Relative biofilm biomass in RPMI-1460 medium at 37°C for 48h. Statistical significance in comparison to the DMSO control group for each strain was determined using one-way ANOVA followed by Tukey’s multiple comparison test for all samples. *P < 0.05, ****P < 0.0001. (TIF) [file ppat.1013519.s006.tif]

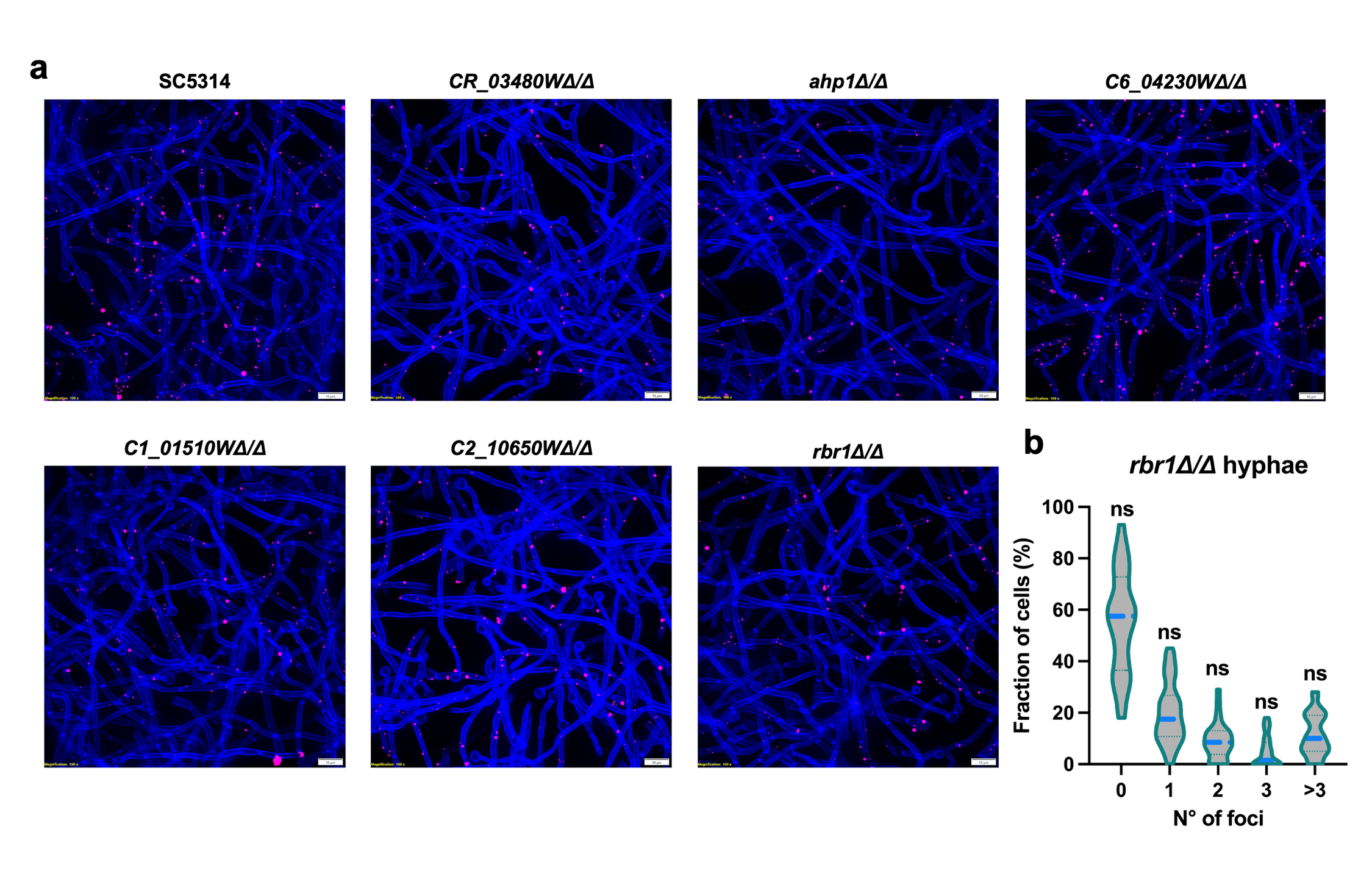

Supplement: S7 Fig — (a) Fluorescence microscopy of C. albicans hyphae of deletion mutants labeled with 1 µM 12mer-Alexa Fluor-647 (purple) and stained with calcofluor white (blue). (b) Foci distribution of C. albicans rbr1∆/∆ hyphae stained with the 12aa-Alexa Fluor-488. Statistical differences were compared to C. albicans SC5314 hyphae by one-way ANOVA followed by Tukey’s multiple comparison test for all samples. (TIF) [file ppat.1013519.s007.tif]
